# Physical Sports Activities and Exercise Addiction during Lockdown in the Spanish Population

**DOI:** 10.3390/ijerph18063119

**Published:** 2021-03-18

**Authors:** Rosendo Berengüí, José María López-Gullón, Salvador Angosto

**Affiliations:** 1 Faculty of Social Sciences and Communication, Catholic University of Murcia, 30107 Guadalupe, Spain; rberengui@ucam.edu; 2 Faculty of Sport Sciences, University of Murcia, 30720 Santiago de la Ribera, Spain; luchamurcia@um.es

**Keywords:** exercise addiction, commitment, health habits, confinement, unsafety

## Abstract

The coronavirus disease 2019 (COVID-19) pandemic has led to the paralysis of the worldwide economy caused by the population’s lockdown to stop the virus spreading, making it difficult to do exercise. The aim of this study is to analyse the commitment to and feeling of insecurity related to practising sport, sports habits and the profile of the Spanish population during lockdown according to the level of addiction to exercise. The sample consisted of 1019 subjects with a mean age of 35 years old. The variables analysed were exercise addiction, commitment to and feeling of insecurity related to sport, and sport habits. Three groups were identified according to their addiction level to exercise: asymptomatic (*n* = 202), symptomatic (*n* = 756), and at risk of addiction (*n* = 61). The main results indicated that a higher addiction level was associated with a higher number of days and time spent on exercise per week. Six percent of the subjects were at addiction risk, and they had a lower feeling of insecurity towards sport. These findings provide information to governments on the need to promote physical activity programmes at home to promote adequate fitness and mental wellbeing in the population.

## 1. Introduction

The coronavirus disease 2019 (COVID-19) pandemic has challenged global health systems in all countries, and it has had a profound impact on the lives of all members of society. In March 2020 in Spain all non-essential industries were shut down indefinitely and public meetings were restricted, forcing millions of people to stay at home as a preventive measure to avoid the spread of coronavirus, due to the risk of propagation at mass gatherings [1,2]. In addition to lockdown, common strategies to mitigate the effects of a pandemic often include the suppression of mass meetings, travel restrictions through border closures and the use of social distancing measures [3].

Naturally, all sports activities also ceased, and in many cases home lockdown led to involuntary physical inactivity. Such inactivity has become a reality for millions of people during the pandemic, as mitigation and containment policies included the closure of sports facilities and leisure infrastructure [4,5].

A third wave of contagion is currently being experienced in Spain, which has forced local governments to take certain measures to halt its advance, such as: (i) closing non-essential businesses or limiting opening hours; (ii) closing social spaces; or (iii) limiting the number of people from the family environment to a maximum of four or six people, depending on the region. Furthermore, local governments also ask the population to carry out self-confinement as a measure to reduce social contacts among the population. In addition to closing sports centres and spaces, this has been forcing people to carry out individual activity outdoors or at home.

Previous studies have found that prolonged stays at home produce radical changes in people’s living habits, not only in terms of physical activity (PA) level, but also in terms of diet, mental health or quality of sleep [6,7,8]. Also, periods of quarantine generally cause negative moods, including frustration, distress, anxiety or boredom [9]. Lockdown can, in many cases, have a negative psychological impact on individuals, increasing the levels of stress or depression resulting from social distancing, as has been observed in past periods of lockdown [10]. Despite the obvious physical consequences, physical inactivity is also associated with symptoms of depression, anxiety and low life satisfaction [11,12].

Therefore, the need for PA within the home is unquestionable, in order to minimise these negative impacts at both levels, physical and psychological [13]. In view of this exceptional situation, a wide spectrum of the population has had difficulty in doing PA at home. Thus, during the period of lockdown there has been an increase in channels that encourage PA online, through different types of platforms such as YouTube or Zoom [14]. These platforms allow real-time interaction between the monitor and participants, in which different activities such as cross-fit, yoga or dance activities could be carried out [15]. Based on this, it appears that the commitment to PA is a key aspect to staying physically active during lockdown, and to having good physical and mental well-being.

In the sports context, commitment is a psychological construct that represents the individual’s determination and desire to continue to engage in PA and sport [16]. Commitment to sport is considered a dynamic and psychological state that can vary over time and its relationship to context [17]. Han and Yang [18] found a relationship between commitment to sport and adherence to PA. A previous study confirms that social support is considered a source of commitment for adult participants [19], with such social support being found in the acceptance of peers [20]. Nevertheless, lack of social support has been shown to be a major source of stress for the people [21].

Concerning commitment, in recent years there has been a growing interest in the analysis of extreme forms of PA, with exercise addiction developing to an excessive extent in certain individuals. The practice of PA and sport is socially accepted, as it is considered a positive contribution for health, even when this exercise is carried out to an excessive extent [22,23]. In certain cases, however, exercise can become an addictive behaviour, being harmful to health, by causing both physical and psychological disorders [24,25].

Exercise addiction is characterised by excessive and obsessive exercise patterns, an abnormal dependence that manifests itself in physiological and/or psychological symptoms [26]. This behavioural process is performed to obtain pleasure or relief from internal discomfort (e.g., stress, anxiety, etc.), and implies the inability to control that behaviour and to persist in it despite the negative consequences it involves [26]. It is generally accepted that exercise addiction shares the essential components with other types of behavioural addictions [24,27,28], specifically: (i) high commitment to desired effects; (ii) prominence, activity becomes the most important thing in the individual’s life and dominates his thoughts, feelings, and behaviour; (iii) conflict, with those around the individual, with other activities, or within the individual himself; (iv) mood change; tolerance, the person requires increasing amounts of exercise to accomplish the desired physical, social, psychological and/or emotional effects; (v) withdrawal symptoms upon cessation of practice; and (vi) relapse, or tendency to repeat the same exercise patterns after a period of time without activity.

Furthermore, for Szabo et al. [28], other characteristics should be considered as possible determinants for their appearance, such as loss of control and lack of commitment over daily activities, loss of control over exercise, risk of self-harm, or denial of the problem, among others. Based on the situation described above concerning the COVID-19 pandemic, it is hypothesised that those subjects who present symptoms that place them at risk of exercise addiction will be less feeling of insecurity toward PA. That is, individuals addicted to exercise may have a greater number of dangerous behaviours, such as not keeping social distance or bad use of the activity zones. The aim of this study is to analyse the degree of commitment and socio-demographic profile of the Spanish population, feeling of insecurity towards sport, and changes in sports habits during the period of lockdown according to the level of exercise addiction.

## 2. Materials and Methods

### 2.1. Participants

The sample was composed of 1019 amateur participants, 52.2% male (*n* = 532) and 47.8% female (*n* = 487) with an average age of 35.31 ± 14.2 years old. The educational level of the participants was 42.0% having completed university or college studies and 30.8% having completed postgraduate studies (master’s degree and/or doctorate). The marital status was approximately half single (51.3%) while those married or cohabiting were 44.0%. Regarding employment status before lockdown, 54.5% were employed or self-employed, 39.8% were students, and only 2.9% were unemployed. In contrast, during lockdown the number of workers fell to 50.4% (working remotely = 37.0%; working face-to-face = 13.4%), while the number of unemployed increased to 13.1% due to employment regulations suffered by partially employed workers and students.

### 2.2. Instrument

The questionnaire was composed of four sections. The first section included questions on commitment to sport, adapted from the runner context developed by Parra-Camacho, Alonso Dos Santos, and González-Serrano [29]. This scale was composed of a total of 11 items distributed into two factors: (i) enthusiasm for sport (six items); and (ii) affliction from sport (five items). The terms related to running were replaced by undertaking sport. The items had a five-point Likert-type response scale (1 = Totally disagree, 2 = Disagree, 3 = Neither agree nor disagree, 4 = Agree, and 5 = Totally agree).

The second section was formed by the Exercise Addiction Inventory (EAI) [30] validated into Spanish by Sicilia et al. [31]. The scale consists of six items preceded by the question “To what extent do you agree with the following statements? The answer was a five-point Likert scale (1 = Strongly disagree; 5 = Strongly agree). Regarding the psychometric properties of the questionnaire, the original study by Terry et al. showed a high internal consistency (Cronbach’s Alpha = 0.84), and analyses of the instrument demonstrated adequate content, concurrent and construct validity. In the Spanish adaptation of Sicilia et al. [31] an alpha value of 0.70 and high level of temporal stability (ICC = 0.92) were obtained, and the structure of the model were invariant across gender. The scores established to determine the level of addiction of the individual establish that the higher the score, the greater the addiction risk to exercise. The score ranges established by the authors of the Instrument were; asymptomatic individuals were those who have a sum of the scores of all items below 12 points; symptomatic individuals, with scores between 13 and 23 points, and finally, individuals with scores above 24 points showed addiction risk [30,31].

The third section is the elaborate ad-hoc scale of feelings of insecurity towards PA. The different perspectives that exist when practicing sports, actively or passively, such as attending sports or fitness centres, as well as professional or amateur sports events, were analysed. These items were distributed into two different periods. We asked about their feeling of insecurity towards carrying out the activities described after lockdown until the appearance of a vaccine, and the feeling of insecurity as from the time that the vaccine existed, through the following phrase; “Safety when carrying out the following physical-sports activities The response scale was a five-point Likert type (1 = None; 2 = Little; 3 = Moderately; 4 = Quite; 5 = Very). Finally, we asked about sociodemographic aspects such as gender, age, marital status, educational level, occupation before and during lockdown, as well as questions related to the practice of physical-sports activity such as the type of activity performed, duration and frequency per week before and during lockdown, and years of sports experience.

### 2.3. Procedure

The procedure had a non-probabilistic design for convenience. The questionnaire was carried out online through the tool of the local university “Surveys”., and it was distributed by specialised forums to which the authors had access, through email to the entire university community and through publication in digital press media of local newspapers. The survey was totally anonymous. The period in which the survey was open was between 24 April and 2 June 2020.

### 2.4. Data Analysis

The data were analysed using the statistical software JASP 0.14 (JASP Team, University of Amsterdam, Amsterdam, The Netherlands). Descriptive statistics were calculated for continuous variables (means and standard deviations) and qualitative variables (frequencies and percentages). An analysis of variance (ANOVA) test was performed to check for differences between groups according to the level of addiction, extracting the Tukey post-hoc, and *t*-test for related samples to observe differences in frequency and practice time between, before and during lockdown. The effect size was calculated according to the guidelines of Dominguez-Lara [32]. The significance level was set at a value of *p* ≤ 0.05.

## 3. Results

### 3.1. Socio-Demographic Profile by Addiction Level

The profile of the amateur participants according to the level of addiction (Table 1) indicated that asymptomatic adult participants represented 19.8% of the total sample, composed of 63.4% of females with an average age of 35.43 ± 15.8. 80. 2% had university or postgraduate studies, were single (51.5%); half were working before lockdown (50.0%) while during lockdown 42.1% were working remotely from home, and most had no one to care for them (64.9%).

Finally, the group at addiction risk was the minority group (6.0%); it was mostly made up of males (62.3%) with an average age of 31.82 ± 13.3 years, half had university or postgraduate studies (52.5%), 57.4% were single and 52.5% worked before the lockdown period.

The group of symptomatic people was made up of 74.2%, the majority were male (55.6%) with an age of 35.56 ± 13.9 years, 72.5% with university education, single (50.8%), 55.8% worked before lockdown and during lockdown 36.0% worked remotely for 32.3% have been unemployed, and lastly, had no one to care for them (64.0%).

However, during the lockdown most were unemployed (44.3%) and 73.8% had no dependents. Comparing the profile of the three groups according to the level of addiction there were statistically significant differences in the variables of gender, educational level, marital status and occupation during lockdown (*p* ≤ 0.05). The effect of variables with statistically significant differences was small with values of Cramer’s V situated between 0.10 and 0.30 point, that is, the variation in the proportions of the variables according to groups is slight.

### 3.2. Sports Habits Profile and Feeling of Insecurity According to Addiction Risk

Table 2 shows the comparative results of the commitment to sport and safety variables according to period and the level of addiction to sport of the participants. The results of the commitment to sport showed that the higher the level of addiction, the higher the scores on both factors, Enthusiasm for Sport and Affliction from Sport. While asymptomatic participants had a low enthusiasm score (M = 2.73 ± 0.9) the group at risk of addiction had a very high value of 4.67 ± 0.4. On the other hand, affliction was higher in the group at addiction risk than the group of asymptomatic participants with scores of 1.87 ± 1.0 and 1.81 ± 0.8, respectively. According to the level of safety towards sports activities, the results showed wide differences between all groups. Asymptomatic participants had a high feeling of insecurity towards participation in physical-sports activities compared to symptomatic subjects, while participants at addiction risk showed safety values higher than three points. The level of safety increased in the period after the existence of the vaccine with respect to the previous period between post-lockdown and the appearance of the vaccine.

Regarding sports habits, asymptomatic subjects indicated that before confinement they engaged in physical activity such as walking or hiking (27.2%) or fitness activities related to running, jogging or cycling (18.8%), with a frequency of 2.88 ± 1.6 days per week and a practice time of 3.58 ± 2.7 h per week. By contrast, this group during confinement increased the frequency of practice to 3.62 days per week, decreasing the duration of PA (M = 2.83 ± 2.5 h per week), performing mainly fitness activities such as yoga, zumba or pilates (30.7%) and fitness conditioning (23.3%). With respect to past experience, they had 6.36 years of previous experience.

The asymptomatic participants group had a higher PA routine than the asymptomatic ones before lockdown, mainly running, jogging or cycling (38.8%) and fitness conditioning (15.9%) with a frequency of 3.93 ± 1.6 days and a duration of 5.31 ± 3.20 h per week. The symptomatic participants had a higher PA routine than the asymptomatic ones before the lockdown, doing mainly running, jogging or cycling (38.8%) and fitness conditioning (15.9%) with a frequency of 3.93 ± 1.6 days and a duration of 5.31 ± 3.20 h per week. Nevertheless, during the lockdown the activities that were carried out primarily were fitness conditioning (39.2%), fitness activities related to yoga or zumba (23.1%) and running or jogging at home (22.9%) during 4.53 ± 1.9 days per week and 4.53 ± 3.1 h per week. The average experience in the practice of PA was 9.73 ± 5.9 years.

The last group of participants at addiction risk before lockdown performed activities such as running, jogging or cycling (45.9%) or fitness conditioning (21.3%), with a frequency of 4.95 ± 1.4 days/week and 6.77 ± 4.10 h per week. However, during lockdown 42.6% did fitness conditioning and 34.4% did running or jogging at home, 5.08 ± 1.8 days and 5.31 ± 3.7 h per week. Previous experience was 9.81 ± 5.5 years. In general, the results showed that as the level of addiction increased, the number of days and hours of weekly PA increased both before and during lockdown, while with respect to the type of activity, the greater the addiction, the greater the proportion of conditioning or running activities. All results showed statistically significant differences (*p* ≤ 0.001), except the affliction from sport factor. The effect of variables with statistically significant differences was medium. The results showed an increase in the number of days of PA and a reduction in the duration of the activity in all the groups. These differences were significant in all cases for each group, except for the frequency of practice of the group at addiction risk.

## 4. Discussion

The aim of this study was to analyse the level of commitment and socio-demographic profile of the Spanish population, feeling of insecurity towards sport and changes in sports habits during the period of lockdown, according to the level of exercise addiction. The COVID-19 pandemic has changed sport at all levels, both elite and grassroots sport. The physical activity habits have been seriously altered, thus seriously altering the well-being of society [4]. The pandemic has affected society as a whole, regardless of socio-economic status, and the virus affects the entire population equally, leaving physical, social and economic consequences [33]. The wider society, from the most vulnerable groups to the richest, is challenged to adapt to this so-called “new normal” [34]. The results obtained in this study provide useful data on the experience of physical-sports practice in the Spanish population during this period.

Firstly, 6% of the participants presented addiction risk, and the percentage of symptomatic participants, 74.2%, was also of concern, as they presented some of the symptoms that lead to addiction. Previous studies had provided very disparate data on prevalence, due to terminological confusion and the assessment instruments used. The systematic review by Marques et al. [35], concluded that people who exercised regularly, showed a prevalence of addiction between 1.9–42.0%, non-sports university students between 3–21.4%, athletes between 1.4–17.0%, and the general population between 0.3–6.4%. In this sense, the data were similar to those found in the present study, with the range confirmed by research using the EAI [30], between 3–5% for the general population and 17% in ultra-marathoners [36,37,38].

The hypothesis is fulfilled; the subjects with exercise addiction obtained significant differences with respect to the other groups, because they were feeling more secure. The results also showed how the subjects at addiction risk and who were symptomatic had a stronger desire to return to practice, in terms of safety towards the practice of PA before and after the existence of the vaccine. As already mentioned, subjects both symptomatic and at addiction risk may engage in a greater number of risky behaviours in relation to COVID-19, and not follow the recommendations of health authorities and the standards applied by the political powers. During the period of lockdown, many cases have been observed in which practitioners broke the rules and went out into the street to perform activities such as running, jogging or cycling.

Other studies that have evaluated the level of safety of the population towards participation in PA in Spain, showed that sports fans would not attend professional leagues or sports events as spectators without prevention measures that respect social distancing and the existence of a COVID-19 vaccine [39]. These authors highlighted that the young population had less uncertainty to attend as spectators, however, they maintained interest in watching television broadcasts. According to the results of this study, where the subjects at addiction risk were the least insecure, they were the youngest in the asymptomatic and symptomatic groups, although there were no significant differences according to age. In the USA, Gentile [40] reported a study of Seton Hall University which indicated that only 13% of fans would feel totally safe attending National Football League games without a vaccine. High levels of exercise addiction can be considered a serious problem with a great impact. Exercise addiction has individual consequences for the subjects, such as conflicts with those around them and with their professional activity, mood changes, tolerance, withdrawal symptoms upon cessation, and relapse [27,28]. Moreover, due to their inability to control such behaviour, these subjects persist with it despite the negative consequences entailed [26,41], because those with more symptoms of addiction may generate more dangerous situations for others. In this sense, they may make dangerous decisions and engage in risky and harmful behaviour, not following the rules set by health authorities. The obsessive component of addiction leads amateurs to pursue excitement and instant rewards; given that they have difficulty delaying gratification, and that thoughtless decision-making prevails, while disregarding the negative consequences of their actions [42,43]. On the other hand, the compulsive component makes amateurs maintain ritualised and stereotypical behaviour [44]. Hence, persistence in practice leads to pathological behaviour, linked to dependence-related harm [45].

The results related to the socio-demographic profile of the participants in our study showed a clear evolution according to the addiction level to exercise. The group of asymptomatic participants was composed mainly of females with an age close to 35 years old, a higher education level and mostly employed. The percentages were equalised in the group of symptom-addicted subjects, and translated in the group of addicted subjects in a large majority of males, with a lower average age, around 31 years old; likewise, a lower proportion of participants with university education, and a high percentage of unemployed during lockdown with respect to the group of asymptomatic subjects. Comparing these results with other similar studies, Marques et al. [46] also had a majority of females among the subjects interviewed in the Brazilian population with an average age of 32.3 years, almost half had university or post-graduate education and were single.

Lesser and Nienhuis [47], in their analysis of the Canadian population, the sample was composed mostly of females, who were married, had completed post-secondary education and were working full time before lockdown. However, 56.8% had changes in their employment status during the period of lockdown, with 32.1% working remotely and 14.7% being dismissed. Concerning their relationship of addiction risk to commitment, this relationship has been observed previously, especially in endurance sports [48], because of the strong demands of these specialties and the high number of hours and sessions dedicated to performance [28,49].

The number of participants in this study needs to be taken into account, as about three out of four exercised individually outdoors, mainly by running. Different studies indicated that, in the endurance modalities, high levels of addiction were registered and the participants continued with the practice in spite of being injured [50], and, moreover, they persisted in spite of the negative consequences caused by not running in the best physical condition, because the reward they obtained was greater than any reward for not doing so [51]. Recent studies indicate that the COVID-19 pandemic has apparently influenced an increase in the active population to be more physically active than before the pandemic, in Spain and the United Kingdom [34,52].

The results on sports habits before and during lockdown were an aspect that has generated concern among governments and experts. This happens because of the changes in the PA levels and sedentarism in the world population, which have had a greater incidence due to the limitations that have occurred in the period of lockdown to avoid the virus spread. The lockdown has been drastically affecting the determining factors of these behaviours at all levels [53]. These results showed that there were significant differences in both the duration and frequency of PA practice according to the level of addiction before and during lockdown, as well as the variation within each group between the two periods. The more addiction to exercise that occurred, the more frequent the practice of PA and the longer the duration while, depending on the period, there was an increase in the frequency of PA practice with a shorter duration during lockdown.

These results coincided with those obtained by Castañeda-Babarro et al. [54], who found that PA decreased significantly during lockdown in the entire Spanish population. These authors observed that vigorous PA and walking time decreased by 16.8% and 58.2%, respectively, while sedentarism increased by 23.8%, with these decreases being greater in males than females. Another study determined that the frequency of PA practice of the Brazilian population was 4.5 days/week, with non-significant variations in frequency before and during lockdown [46].

A recent study in the German population also found that there was a reduction in the level of PA before and during lockdown, where inactive persons increased by 20.1%, and those active decreased their activity time considerably [55]. Within the Canadian population, Lesser and Nienhuis [47] found that 63.4% of the population analysed did not meet the minimum recommendations for weekly PA hours. A 40.5% of this group reduced their PA level during lockdown while in the active group it was 22.4%. The type of activity performed by inactive persons was walking around the neighbourhood, and in active persons it was jogging or running at home.

Reductions in PA levels and increased sedentariness have previously been corroborated in populations that have suffered similar lockdown due to adverse hot or cold weather conditions, where the weather is a barrier to getting outdoors, especially in older adults [56,57,58]. In turn, the physically active population tends to have fewer experiences of stress, depression, or anxiety than inactive individuals [59]. In order to stay active and not spend as much time sitting in these situations, Loh et al. [60] recommend that one way to break up sitting time is to take a two-minute walk every 20–30 min seated.

Finally, the results on the type of PA performed determined that before lockdown the main activities of the different groups according to the addiction level were walking or hiking, running or cycling. The main activities during lockdown were those related to fitness conditioning and fitness activities (e.g., pilates, yoga, zumba, etc.). There were significant differences in the activities by group. The group of participants at addiction risk showed a high percentage of running or cycling activities in the home during the lockdown period compared to the other two groups. Marques et al. [39], indicated that the Brazilian population increased the practice of activities related to conditioning by 8.7%, walking or running increased by 7.4% and functional training increased by 44.9% during lockdown. Schwendinger and Pocecco [61] indicated that an ideal activity to perform at home may be functional training, due to the short duration of intervals and minimal use of equipment.

This study found several limitations. Firstly, as it this was convenience sampling, the results cannot be generalised to the whole Spanish population or other countries. Secondly, there was a temporal limitation of the data collection, since the data collection took place during the week prior to the end of lockdown with the aim of creating a PA practice habit to evaluate during the following weeks of forced lockdown at home. Thirdly, one limitation associated with the exercise addiction instrument may be its rating scale. Although any classification can be considered arbitrary, the study by Terry et al. [30] proposed that the cut-off score for individuals considered at-risk of exercise addiction was 24. This cut off represents those individuals with scores in the top 15% of the total scale score. Taking these cut-off points into account, in the study of Sicilia et al. [31] a 14.9% of participants obtained a total score equal to or higher than 24 in the EAI and were classified as being at risk of exercise addiction. Another limitation of this study has been the impossibility of determining the degree of compliance with the minimum PA recommendations as there was no difference between type of activity, vigorous or moderate, as well as sedentary attitudes during lockdown. A final limitation may be that the population on which the survey was disseminated had a profile of sports people; although it was also published on the web by regional generalist media, it is more likely that active people were more predisposed to perform it than sedentary people.

## 5. Conclusions

The main findings of this study indicate that, in the search for safe practice and responsible use of sports spaces and facilities, in the new times and current and future contexts, it is necessary to go deeper into this area of analysis with a view to optimal intervention. Although the numbers of people at addiction risk in this and other studies are relatively low, it is verified that symptomatic practitioners are the great majority. It is possible that the development and validation of instruments and the uniformity of diagnostic criteria will increase these data [62]. However, at the same time the detection of precise cases of help and those who engage in behaviour that is a risk to others and to themselves are always undertaken with a view to guiding sports activities in the direction of the health of all those involved [63].

The socio-demographic profile of the different groups indicated that the asymptomatic subjects are mainly female, with an average age of 35 years old, single, with a university education and working, while the subjects at addiction risk are, proportionally, mostly males of 31 years old with a university education, single and working. Taking into account the differences in occupation before and during lockdown, the results indicate that the number of unemployed people increased and most of them work remotely from home. Finally, sports habits indicate the existence of significant differences according to the addiction level and according to the period before and during lockdown, both in frequency and duration of PA, except for the group at addiction risk, which varies greatly in the frequency of PA.

National and regional governments should be responsible for implementing measures that encourage initiatives to carry out PA at home, complying with the minimum recommendations that will enable the population to remain physically active, without the need to use equipment that may create barriers to practice and prevent the adoption of sedentary attitudes. Currently there are many tools and possibilities to bring PA programs to the vast majority of the population through the use of new technologies that allow us not only to record the PA that is carried out for later analysis, but online platforms that allow a live interaction between specialists and subjects can encourage a healthier habit through obtaining feedback. The practice of PA not only will allow us to reduce sedentary behaviour, but will also favour better mental health and nutrition.

Maintaining high levels of collective well-being during a pandemic is crucial. Hope and optimism are key to coping and persevering, while frustration and dissatisfaction can erode commitment to public health measures and containment policies. Therefore, supporting people to stay active should be considered an integral part of pandemic-related public health measures [4].

## Figures and Tables

**Table 1:** Sociodemographic profile of the participants by addiction level.

Variables	Asymptomatic	Symptomatic	Addiction Risk
	(*n* = 202)	(*n* = 756)	(*n* = 61)
	M (SD)	M (SD)	M (SD)
Age	35.43 (15.8)	35.56 (13.9)	31.82 (13.3)
(F = 1.96(2); *p* = 0.141; η^2^ = 0.004)			
	N (%)	N (%)	N (%)
Gender * (x^2^ = 25.5(2); *p* ≤ 0.001; V = 0.158)			
Male	74 (36.6)	420 (55.6)	38 (62.3)
Female	128 (63.4)	336 (44.4)	23 (37.7)
Education level * (x^2^ = 43.3(12); *p* ≤ 0.001; V = 0.146)			
Elementary studies	3 (1.5)	44 (5.8)	12 (19.7)
High School/Vocational education	36 (17.8)	156 (20.6)	16 (26.2)
Graduated or posgraduated estudies	162 (80.2)	548 (72.5)	32 (52.5)
Other	1 (0.5)	8 (1.1)	1 (1.6)
Marital Status (x^2^ = 5.57(6); *p* = 0.473; V = 0.052)			
Single	104 (51.5)	384 (50.8)	35 (57.4)
Married or cohabitating	86 (42.6)	341 (45.1)	21 (34.4)
Divorced or separated	11 (5.4)	29 (3.8)	5 (8.2)
Widowed	1 (0.5)	2 (0.3)	-
Occupation BL (x^2^ = 6.39(6); *p* = 0.381; V = 0.056)			
Unemployed	3 (1.5)	24 (3.2)	3 (4.9)
Employed	101 (50.0)	422 (55.8)	32 (52.5)
Retired	5 (2.5)	22 (2.9)	1 (1.6)
Student	93 (46.0)	288 (38.1)	25 (41.0)
Occupation DL * (x^2^ = 18.7(8); *p* = 0.016; V = 0.096)			
Unemployed	50 (24.8)	244 (32.3)	27 (44.3)
Remote employee	85 (42.1)	272 (36.0)	20 (32.8)
On-site employee	19 (9.4)	112 (14.8)	6 (9.8)
Retired	5 (2.5)	22 (2.9)	1 (1.6)
Student	43 (21.3)	106 (14.0)	7 (11.5)

Note: BC: before lockdown. DL: during lockdown. * *p* ≤ 0.05. η^2^: squared eta; between η^2^ < 0.01 trivial effect, between 0.01< η^2^ <0.06 small effect, between 0.06 < η^2^ < 0.14 medium effect, and η^2^ > 0.14 significant effect. V: Cramer’s V; V < 0.10: irrelevant effect, between 0.10 < V< 0.30: small effect, between 0.30 < V< 0.50: moderate effect, and V > 0.50 large effect.

**Table 2:** Sports profile of participants by exercise addiction level.

Variables	Asymptomatic	Symptomatic	Addiction Risk
	(*n* = 202)	(*n* = 756)	(*n* = 61)
	M (SD)	M (SD)	M (SD)
Enthusiasm * (F = 317.92(2); *p* ≤ 0.001; η^2^ = 0.385)	2.73 (0.9)	4.02 (0.7)	4.67 (0.4)
Affliction	1.81 (0.8)	1.76 (0.8)	1.87 (1.0)
(F = 0.84(2); *p* = 0.431; η^2^ = 0.002)			
Feeling of insecurity ABV *	1.72 (0.8)	2.41 (1.0)	3.33 (1.1)
(F = 76.14(2); *p* ≤ 0.001; η^2^ = 0.130)			
Feeling of insecurity AAV *	2.14 (1.0)	3.00 (1.1)	3.60 (1.1)
(F = 69.82(2); *p* ≤ 0.001; η^2^ = 0.121)			
Frequency of PA practise BL *	2.88 (1.6)	3.93 (1.6)	4.95 (1.4)
(F = 54.97(2); *p* ≤ 0.001; η^2^ = 0.098)			
Duration of PA practise BL *	3.58 (2.7)	5.31 (3.2)	6.77 (4.1)
(F = 33.68(2); *p* ≤ 0.001; η^2^ = 0.062)			
Frequency of PA practise DL *	3.62 (2.2)	4.53 (1.9)	5.08 (1.8)
(F = 21.45(2); *p* ≤ 0.001; η^2^ = 0.041)			
Duration of PA practise DL *	2.83 (2.5)	4.53 (3.1)	5.31 (3.7)
(F = 28.73(2); *p* ≤ 0.001; η^2^ = 0.054)			
Sports Experience *	6.36 (6.4)	9.73 (5.9)	9.81 (5.5)
(F = 26.05(2); *p* ≤ 0.001; η^2^ = 0.049)			
	N (%)	N (%)	N (%)
Type of Physical Activity PL * (x^2^ = 117.96(10); *p* ≤ 0.001; V = 0.241)			
Walking or hiking	55 (27.2)	56 (7.4)	-
Running, jogging, cycling	38 (18.8)	293 (38.8)	28 (45.9)
Fitness conditioning	23 (11.4)	120 (15.9)	13 (21.3)
Fitness activities (yoga. pilates. zumba. etc.)	32 (15.8)	84 (11.1)	1 (1.6)
Other activities	40 (19.8)	190 (25.1)	19 (31.2)
Nothing	14 (6.9)	13 (1.7)	-
Type of Physical Activity DL * (x^2^ = 58.24(10); *p* ≤ 0.001; V = 0.169)			
Walking or hiking	22 (10.9)	39 (5.2)	2 (3.3)
Running, jogging, cycling	37 (18.3)	173 (22.9)	21 (34.4)
Fitness conditioning	47 (23.3)	296 (39.2)	26 (42.6)
Fitness activities (yoga. pilates. zumba. etc.)	62 (30.7)	175 (23.1)	8 (13.1)
Other activities	5 (2.5)	36 (4.8)	2 (3.3)
Nothing	29 (14.4)	37 (4.9)	2 (3.3)

Note: ABV: Activities before vaccine; AAV: Activities after vaccine; * Significant differences between all groups; BL: before lockdown. DL: during lockdown. * Significant differences between all groups (*p* ≤ 0.001). η^2^: squared eta; between η^2^ < 0.01 trivial effect, between 0.01< η^2^ <0.06 small effect, between 0.06 < η^2^ < 0.14 medium effect, and η^2^ > 0.14 significant effect. V: Cramer’s V; V < 0.10: irrelevant effect, between 0.10 < V< 0.30: small effect, between 0.30 < V< 0.50: moderate effect, and V > 0.50 large effect.

## Data Availability

Data sharing not applicable.

## References

[1] Majumdar B., Naha S. (2020). Live sport during the COVID-19 crisis: Fans as creative broadcasters. Sport Soc..

[2] Memish Z.A., Steffen R., White P., Dar O., Azhar E.I., Sharma A., Zumla A. (2019). Mass gatherings medicine: Public health issues arising from mass gathering religious and sporting events. Lancet.

[3] Anderson R., Heesterbeek H., Klinkenberg D., Hollingsworth T.D. (2020). How will country-based mitigation measures influence the course of the COVID-19 epidemic?. Lancet.

[4] Mutz M. (2020). Forced adaptations of sporting behaviours during the Covid-19 pandemic and their effects on subjective well-being. Eur. Soc..

[5] Shahidi S.H., Stewart J., Hassani F. (2020). Physical activity during COVID-19 quarantine. Acta Paediatrica.

[6] Altena E., Baglioni C., Espie C., Ellis J., Gavriloff D., Holzinger B., Schlarb A., Frase L., Jernelöv S., Riemann D. (2020). Dealing with sleep problems during home confinement due to the COVID-19 outbreak: Practical recommendations from a task force of the European CBT-I Academy. J. Sleep Res..

[7] Jiménez-Pavón D., Carbonell-Baeza A., Lavie C.J. (2020). Physical exercise as therapy to fight against the mental and physical consequences of COVID-19 quarantine: Special focus in older people. Prog. Cardiov. Dis..

[8] (2020). World Health Organization Mental Health. Mental Health and Psychosocial Considerations during the COVID-19 Outbreak.

[9] Brooks S.K., Webster R.K., Smith L., Woodland L., Wessely S., Greenberg N., Rubin G.J. (2020). The psychological impact of quarantine and how to reduce it: Rapid review of the evidence. Lancet.

[10] Hawryluck L., Gold W.L., Robinson S., Pogorski S., Galea S., Styra R. (2004). SARS control and psychological effects of quarantine, Toronto, Canada. Emerg. Infect. Dis..

[11] Pengpid S., Peltzer K. (2019). Sedentary behaviour, physical activity and life satisfaction, happiness and perceived health status in University students from 24 Countries. Int. J. Environ. Res. Public Health.

[12] Rebar A.L., Stanton R., Geard D., Short C., Duncan M.J., Vandelanotte C. (2015). A meta-meta-analysis of the effect of physical activity on depression and anxiety in non-clinical adult populations. Health Psychol. Rev..

[13] Hammami A., Harrabi B., Mohr M., Krustrup P. (2020). Physical activity and coronavirus disease 2019 (COVID-19): Specific recommendations for home-based physical training. Manag. Sport Leis..

[14] Ng K. (2020). Adapted physical activity through COVID-19. Europ. J. Adap. Phys. Activ..

[15] Nyenhuis S.M., Greiwe J., Zeiger J.S., Nanda A., Cooke A. (2020). Exercise and Fitness in the age of social distancing during the COVID-19 Pandemic. J. Allerg. Clin. Immun. Pract..

[16] Scanlan T.K., Russell D.G., Scanlan L.A., Klunchoo T.J., Chow G.M. (2013). Project on elite athlete commitment (PEAK): IV. Identification of new candidate commitment sources in the sport commitment model. J. Sport Exerc. Psychol..

[17] Williams L. (2013). Commitment to sport and exercise: Re-examining the literature for a practical and parsimonious model. J. Prev. Med. Public Health.

[18] Han K.S., Yang C.H. (2018). The relationship between yacht school participants’ sport commitment and exercise adherence. Indian J. Public Health Res. Dev..

[19] Weiss M.R., Kimmel L.A., Smith A.L. (2001). Determinants of sport commitment among junior tennis players: Enjoyment as a mediating variable. Pediat. Exerc. Sci..

[20] Crone E.A., Güroǧlu B., Ochsner K.N., Kosslyn S.M. (2014). Development of emotion and social reasoning in adolescence. The Oxford Handbook of Cognitive Neuroscience.

[21] DeFreese J.D., Smith A.L. (2013). Teammate social support, burnout, and self-determined motivation in collegiate athletes. Psychol. Sport Exerc..

[22] Forrest L.N., Smith A.R., Fussner L.M., Dodd D.R., Clerkin E.M. (2016). Using implicit attitudes of exercise importance to predict explicit exercise dependence symptoms and exercise behaviours. Psychol. Sport Exerc..

[23] Lichtenstein M.B., Hinze C.J., Emborg B., Thomsen F., Hemmingsen S.D. (2017). Compulsive exercise: Links, risks and challenges faced. Psychol. Res. Behav. Manag..

[24] Griffiths M.D. (2002). Gambling and Gaming Addictions in Adolescence.

[25] Márquez S., De la Vega R. (2018). La adicción al ejercicio: Un trastorno emergente de la conducta. Nutr. Hospit..

[26] Berengüí R., Pelegrín A., Berengüí R., López-Walle J.M. (2018). Problemas asociados a la práctica deportiva [Problems associated with practising sport]. Introduction to Sports Psychology.

[27] Sussman S., Sussman A.N. (2011). Considering the Definition of Addiction. Int. J. Environ. Res. Public Health.

[28] Szabo A., Griffiths M.D., Demetrovics Z., Preedy V.R. (2016). Exercise addiction. Neuropathology of Drug Addictions and Substance Misuse, Volume 3: General Processes and Mechanisms, Prescription Medications, Caffeine and Areca, Polydrug Misuse, Emerging Addictions and Nondrug Addictions.

[29] Parra-Camacho D., Alonso Dos Santos M., González-Serrano M.H. (2020). Amateur Runners’ Commitment: An Analysis of Sociodemographic and Sports Habit Profiles. Int. J. Environ. Res. Public Health.

[30] Terry A., Szabo A., Griffiths M.D. (2004). The Exercise Addiction Inventory: A new brief screening tool. Addict. Res. Theor..

[31] Sicilia A., Alías-García A., Ferriz R., Moreno-Murcia J.A. (2013). Spanish adaptation and validation of the Exercise Addiction Inventory (EAI). Psicothema.

[32] Dominguez-Lara S. (2018). Effect size, a quick guide. Educac. Méd..

[33] Ali S., Asaria M., Stranges S. (2020). COVID-19 and inequality: Are we all in this together?. Canad. J. Public Health.

[34] Grix J., Brannagan P.M., Grimes H., Neville R. (2020). The impact of Covid-19 on sport. Int. J. Sport Polic. Polit..

[35] Marques A., Peralta M., Sarmento H., Loureiro V., Gouveia E.R., de Matos M.G. (2018). Prevalence of risk for Exercise Dependence: A Systematic Review. Sports Med..

[36] Griffiths M.D., Szabo A., Terry A. (2005). The exercise addiction inventory: A quick and easy screening tool for health practitioners. Br. J. Sports Med..

[37] Monok K., Berczik K., Urbán R., Szabo A., Griffiths M.D., Farkas J., Magi A., Eisinger A., Kurimai T., Kökönyei G. (2012). Psychometric properties and concurrent validity of two exercise addiction measures: A population wide study. Psychol. Sport Exerc..

[38] Szabo A., De la Vega R., Ruiz-Barquín R., Rivera O. (2013). Exercise addiction in Spanish athletes: Investigation of the roles of gender, social context and level of involvement. J. Behav. Addict..

[39] Vegara-Ferri J.M., Carboneros M., Deliautaite K., Díaz-Suárez A., López-Gullón J.M. (2020). Fan’s perspective on professional leagues and sporting events during COVID-19 confinement period. J. Hum. Sport Exerc..

[40] Gentile R. (2020). Nearly 3 of 4 Americans Say They Won’t Attend Games without Coronavirus Vaccine Developed. Seton Hall Sports Poll. http://blogs.shu.edu/sportspoll/2020/04/09/nearly-3-of-4-americans-say-they-wont-attend-games-without-coronavirus-vaccine-developed/.

[41] Sellman D. (2016). Behavioural health disorders rather than behavioural addictions. Australian and New Zealand. J. Psych..

[42] Cook B.J., Hausenblas H.A., Tuccitto D., Giacobbi P. (2011). Eating disorders and exercise: A structural equation modeling analysis of a conceptual model. Eur. Eat. Disord. Rev..

[43] Grant J.E., Potenza M.N. (2006). Compulsive aspects of impulse-control disorders. Psychiatr. Clin..

[44] Freimuth M., Moniz S., Kim S.R. (2020). Clarifying exercise addiction: Differential diagnosis, co-occurring disorders, and phases of addiction. Int. J. Environ. Res. Public Health.

[45] Tunney R.J., James R.J. (2017). Criteria for conceptualizing behavioural addiction should be informed by the underlying behavioural mechanism. Addiction.

[46] Marques J., Andrade R., Gomes L., Landeira-Fernández J., Filgueiras A. (2020). Effects of physical activity and exercise on well-being in the context of the Covid-19 pandemic. MedRxiv.

[47] Lesser I.A., Nienhuis C.P. (2020). The Impact of COVID-19 on Physical Activity Behavior and Well-Being of Canadians. Int. J. Environ. Res. Public Health.

[48] Lu F.J., Hsu E.Y., Wang J.M., Huang M.Y., Chang J.N., Wang C.H. (2012). Exercisers’ identities and exercise dependence: The mediating effect of exercise commitment. Percept. Motor Skills.

[49] Murray A.L., McKenzie K., Newman E., Brown E. (2013). Exercise identity as a risk factor for exercise dependence. British J. Health Psychol..

[50] Martin L.E., Sisante J.F., Wilson D.R., Moody A.A., Savage C.R., Billinger S.A. (2017). Pilot study of endurance runners and brain responses associated with delay discounting. Int. J. Exerc. Sci..

[51] Nogueira A., Molinero O., Salguero A., Márquez S. (2018). Exercise addiction in practitioners of endurance sports: A literature review. Front. Psychol..

[52] Angosto S., Berengüí R., Vegara-Ferri J.M., López-Gullón J.M. (2020). Motives and Commitment to Sport in Amateurs during Confinement: A Segmentation Study. Int. J. Environ. Res. Public Health.

[53] Hall G., Laddu D.R., Phillips S.A., Lavie C.J., Arena R. (2020). A tale of two pandemics: How will COVID-19 and global trends in physical inactivity and sedentary behavior affect one another?. Prog. Cardiov. Dis..

[54] Castañeda-Babarro A., Arbillaga-Etxarri A., Gutiérrez-Santamaria B., Coca A. (2020). Impact of COVID-19 confinement on the time and intensity of physical activity in the Spanish population. Res. Sq..

[55] Mutz M., Gerke M. (2020). Sport and exercise in times of self-quarantine: How Germans changed their behaviour at the beginning of the Covid-19 pandemic. Int. Rev. Sociol. Sport.

[56] Arnardottir N.Y., Oskarsdottir N.D., Brychta R.J., Koster A., Van Domelen D.R., Caserotti P., Eiriksdottir G., Sverrisdottir J.E., Johannsson E., Launer L.J. (2017). Comparison of Summer and Winter Objectively Measured Physical Activity and Sedentary Behavior in Older Adults: Age, Gene/Environment Susceptibility Reykjavik Study. Int. J. Environ. Res. Public Health.

[57] Jones G.R., Brandon C., Gill D.P. (2017). Physical activity levels of community-dwelling older adults are influenced by winter weather variables. Arch. Geront. Geriat..

[58] Nakashima D., Kimura D., Watanabe H., Goto F., Kato M., Fuji K., Kasuya E., Tomiyama N., Hasegawa R. (2019). Influence of seasonal variations on physical activity in older people living in mountainous agricultural areas. J. Rural. Med..

[59] Chekroud S.R., Gueorguieva R., Zheutlin A.B., Paulus M., Krumholz H.M., Krystal J.H., Chekroud A.M. (2019). Association between physical exercise and mental health in 12 million individuals in the USA between 2011 and 2015: A cross-sectional study. Lancet Psychiatry.

[60] Loh R., Stamatakis E., Folkerts D., Allgrove J.E., Moir H.J. (2019). Effects of Interrupting Prolonged Sitting with Physical Activity Breaks on Blood Glucose, Insulin and Triacylglycerol Measures: A Systematic Review and Meta-analysis. Sports Med..

[61] Schwendinger F., Pocecco E. (2020). Counteracting Physical Inactivity during the COVID-19 Pandemic: Evidence-Based Recommendations for Home-Based Exercise. Int. J. Environ. Res. Public Health.

[62] Nogueira A., Salguero A., Márquez S. (2017). Adicción a correr: Una revisión desde sus inicios hasta la actualidad [Running addiction: A review from its beginnings to the present]. Rev. Psicol. Aplic. Deport Ejerc. Fís..

[63] Smith D., Wright C., Winrow D. (2010). Exercise dependence and social physique anxiety in competitive and non-competitive runners. Int. J. Sport Exerc. Psychol..

